# A hundred-year-old insight into the gut microbiome!

**DOI:** 10.1186/1757-4749-1-21

**Published:** 2009-12-07

**Authors:** Ramy Karam Aziz

**Affiliations:** 1 Department of Microbiology and Immunology, Faculty of Pharmacy, Cairo University, 11562 Cairo, Egypt

## Abstract

As the National Institutes of Health-funded Human Microbiome Project enters its second phase, and as a major part of this project focuses on the human gut microbiome and its effects on human health, it might help us to travel a century back in time and examine how microbiologists dealt with microbiome-related challenges similar to those of the 21^st ^century using the tools of their time. An article by Arthur I. Kendall, published in *The Journal of Biological Chemistry *in November 1909 (Some observations on the study of the intestinal bacteria *J Biol Chem *1909, 6:499-507), offers a visionary insight into many of today's hot research questions.

## Why the gut?

When *Gut Pathogens *was launched earlier this year [1], I was among those surprised that a scientific journal would focus entirely on the pathogens that colonize, parasitize, and potentially damage a single mammalian system: the alimentary canal or ***the gut***. This surprise, however, would quickly vanish on considering the number of microorganisms that inhabit this single body system. For example, an estimated 10 to 100 trillion (10^13^-10^14^) bacteria and archaea inhabit the human gut [2-4], and this suggests 10^14^-10^15 ^bacteriophages associated with them [5,6], not to mention other viruses and other parasitic eukaryotic microbes. While most of the aforementioned members of the human microbiota are not classically described as pathogens, their role in pathogenesis is being (re)discovered [7]. Diseases are being increasingly attributed to multiple organisms and microbial consortia [8,9], to shifts in the composition of the so-called 'normal' flora (e.g., [10-14]), and to disturbances in the balance between the microbial flora and the host's immune system (e.g., [15-18]). *Gut Pathogens*, being equally interested in the health and disease of the alimentary canal, and in the role and biology of its invisible inhabitants [1], certainly comes at the right time to bring a well-needed emphasis on this body system and its microbiome.

Concomitant with the evolution of how we view the microbial role in the gut's health and disease came a revolution in sequencing technology, whereby microbial genomes [19] and metagenomes [20] are being sequenced and made publicly available with unprecedented speed and accuracy. These revolutions in thought and technology have opened an entirely new arena in the research of human health and disease. The Human Genome Project (HGP) has now cleared the way to its sequel, the Human Microbiome Project (HMP), launched by the US National Institutes of Health (NIH) in 2007 [21]. HMP aims at sequence-based determination of the composition and function of the gut microflora, most of which was missed by culture-based studies [9,22].

## Old is new

**Figure 1:**
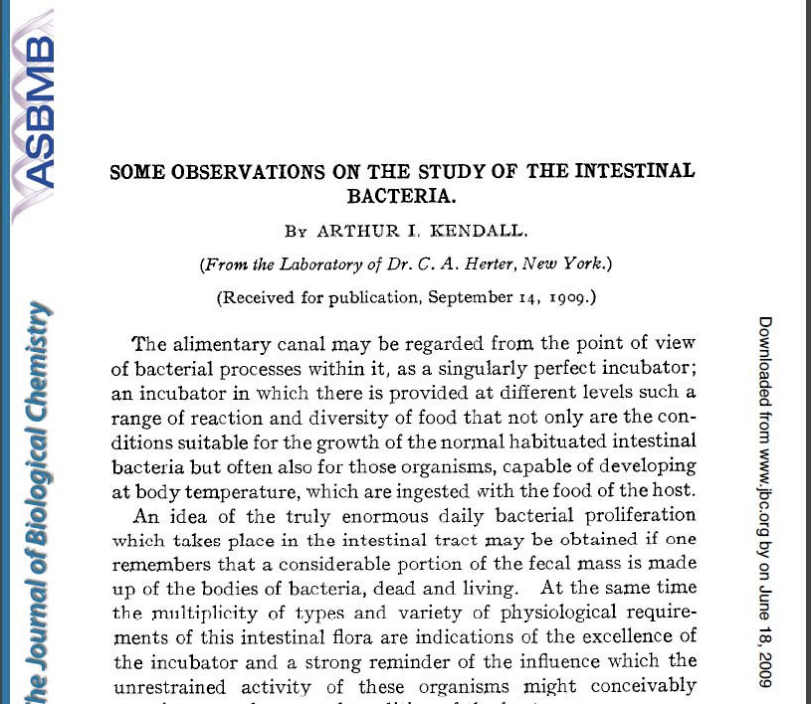
**A screenshot of the first page of the restored electronic version of Kendall's article **[24], **discussed in this *Commentary***. The article can be downloaded from TinyURL: http://tinyurl.com/ydpm8rc[25].

But, is this arena completely *new*? A recent discussion about bacteriocentrism [23] led me to discover a hidden gem, written by Arthur I. Kendall and published by *The Journal of Biological Chemistry *(JBC) in November 1909, a hundred years ago [24]! This article (Fig. 1), which probably includes the first documented occurrence of the word '*bacteriocentric*' in scientific literature, also addresses many of the current concerns of the HMP, not including^_^of course^_^the sequence-based methodology. Kendall's article is a gentle reminder that, while today's technology offers us tremendous advantage over the past decades and centuries, this should in no way lead us to the arrogance of underestimating or overlooking the product of human thought at any age. Darwin's works, which the scientific community is celebrating this year, are another good reminder.

The article's full text is freely available as a PDF file (TinyURL: http://tinyurl.com/ydpm8rc) thanks to the open access policy adopted by JBC (URL: http://www.jbc.org), and I invite *Gut Pathogens *readers to download it [25], read it, and enjoy a glimpse into the microbiological research scene 100 years ago (Fig. 1). The article concentrates on the effect of diet on the microbiome and the effect of the microbiome on human health, with emphasis on biochemistry and metabolites rather than on cataloguing microbial taxa [24]. These are recurrent themes in today's metagenomic and microbiome research [26-28] and are very pertinent to some of the specific aims of the HMP [21], notably at its second phase [29].

Without further ado, I suggest you read some excerpts of the article discussing these recurrent themes.

### Excerpts of the article (I): (emphasis mine)

"The alimentary canal may be regarded from the point of view of bacterial processes within it, as a singularly perfect incubator; an incubator in which there is provided at different levels such a range of reaction and diversity of food that not only are the conditions suitable for the growth of the normal habituated intestinal bacteria but often also for those organisms, capable of developing at body temperature, which are ingested with the food of the host.

An idea of the truly enormous daily bacterial proliferation which takes place in the intestinal tract may be obtained if one remembers that **a considerable portion of the fecal mass is made up of the bodies of bacteria, dead and living**. At the same time the multiplicity of types and variety of physiological requirements of this intestinal flora are indications of the excellence of the incubator and **a strong reminder of the influence which the unrestrained activity of these organisms might conceivably exercise upon the general condition of the host**." (p. 499)

"The very importance of these discoveries has been a potent factor in diverting attention from the studies of the normal intestinal flora with its wealth of problems relating to the principles which govern the activity of these bacteria. Even at the present time the sequence of events which permits the establishment of these exogenous invaders in the alimentary canal and the exact conditions through which they are able not only to extend and maintain themselves but even to replace wholly or in part the normal flora, are unknown." (p. 499-500)

"The lack of appreciation of this fundamental difference which exists between the relatively inert pathogens and the chemical activity of the more important types of the normal intestinal flora, together with the notoriety that attaches to the former, explains the unprogressive attitude which has characterized many researches on intestinal bacteriology." (p. 500)

"This 'bacteriocentric' conception is not illogical when one is dealing with the exogenous pathogens mentioned above, but it is unproductive of definite results when it is applied in its unmodified form to the study of the normal intestinal flora. **It is becoming more and more evident that the problem of intestinal bacteriology must be approached from the dynamical rather than from the cultural standpoint**.

Dr. Theobald Smith^1 ^has stated the case admirably in the following terms: '**It is what bacteria do rather than what they are that commands attention**, since our interest centers in the host rather than in the parasite.'

^1 ^Theobald Smith: Some Problems in the Life-history of Pathogenic Microörganisms, Amer. Med., viii, pp. 711-718, 1904." (p. 500-501)

## Are we what we eat?

Today, we are still intrigued by the vast inter-individual differences in the gut microflora [26,30]. While humans differ by a minute fraction of their inherited nucleic acids [31], the additional genomes^_^or rather metagenomes^_^that they acquire a few months after birth [30,32] carry major differences that distinguish each individual, but are surprisingly stable over time within an individual (unless diet, immune system, health condition, or medication leads to a temporary or permanent restructuring of these communities [7,33]). As we establish the effect of human genetics on bacterial diseases [34,35], we are beginning to unravel how such genetic factors play dramatic effects on gut colonization by different microbial taxa [30,36]. More interestingly, we are just scratching the surface of the anthropological, cultural, and environmental effects on this major part of the human normal flora. Diet, on which Kendall focuses in this article, varies tremendously even among genetically similar individuals. In highly populated countries (e.g., India and China), where societies are separated by complex cultural, religious, linguistic, and geographic boundaries [26], all these separating factors may affect diet significantly and subsequently alter the composition and biochemistry of the gut inhabitants. A hundred years after Kendall's article [24], HMP investigators have also found that not only what we eat affects our microbiome, but that our microbiome might influence what we eat (chocolate craving [37]) and how we assimilate it (obesity) [13,14,38].

Now that we have the possibility of studying the gut microbiome by DNA-based methods, the paradigm shift advanced by Kendall is still well needed, even after 100 years. "It is what bacteria do rather than what they are" [in [24]] that counts, whether our focus is on the mammalian host or on the entire ecosystem in which single-celled organisms coexist with multicellular tissues and organs.

I conclude this *Commentary *by letting you read more excerpts from Kendall's article [24] pertaining to dietary effects on the intestinal microflora, and their detection with what today we call metabolites profiling, metabolomics [33], or metabonomics [28].

### Excerpts of the article (II)

"Having determined by experiment that a given diet (for example, simple protein) is associated with a definite type of bacterial activity, and that coincidentally certain of these indicators are present in the urine of the host, it becomes a relatively simple matter to isolate individual strains of this fecal flora which will reproduce, either alone or symbiotically with other strains, these same end products." (p. 503)

"For example, if the experimental animal is on a carbohydrate regimen, the presence or absence of growth in protein media will indicate the presence or absence of proteolytic bacteria, since the acidophilic organisms do not grow well in these media and cannot, therefore, inhibit the growth of these organisms. Conversely, with a protein diet, the presence or absence of acidophiles may be determined by inoculating the mixed fecal flora into acid dextrose broth, which is unfavorable for the development of the proteolytic types. These determinations may be made roughly quantitative for the different types by inoculating definite amounts of the mixed fecal flora into appropriate media.

The end products of bacterial activity which appear in the urine are important for two reasons: they indicate the types of bacterial activity in the intestinal tract, and their reproduction in artificial media by pure cultures derived from the intestinal flora furnish strong presumptive evidence of the participation of these organisms in the process" (p. 507).
